# Diagnostic Criteria and Clinical Outcomes in Sarcopenia Research: A Literature Review

**DOI:** 10.3390/jcm7040070

**Published:** 2018-04-08

**Authors:** Alex Han, Steven L. Bokshan, Stephen E. Marcaccio, J. Mason DePasse, Alan H. Daniels

**Affiliations:** 1 Department of Orthopedic Surgery, Alpert Medical School of Brown University, 100 Butler Drive, Providence, RI 02906, USA; alex_han@brown.edu (A.H.); stephen_marcaccio@brown.edu (S.E.M.); jmdepasse@gmail.com (J.M.D.); 2 Department of Orthopaedics, Division of Spine Surgery—Adult Spinal Deformity Service, Warren Alpert Medical School of Brown University, Providence, RI 02906, USA; alandanielsmd@gmail.com

**Keywords:** sarcopenia, muscle mass, atrophy, aging, review

## Abstract

By the sixth decade of life, nearly one quarter of the population has substantial muscle atrophy, or sarcopenia. Despite the creation of a standardized definition of sarcopenia by the European Working Group on Sarcopenia in Older People, variability may exist in the diagnostic criteria utilized for clinical sarcopenia research. The primary objectives of this review were to characterize diagnostic criteria used for measurement of sarcopenia in original studies, and to describe associations between sarcopenia and important clinical outcomes. We performed a literature review of the term “sarcopenia” in PubMed. Inclusion criteria were English language, original data, a clear and specific definition for diagnosing sarcopenia, and the analysis of sarcopenia’s effect on a clinical outcome. A total of 283 studies met inclusion criteria. More than half of the included sarcopenia investigations were level IV studies (54.1%), while 43.1% provided level II evidence. Under one third (27.6%) of studies examined sarcopenia with regard to surgical outcomes. In terms of diagnostic criteria for sarcopenia, 264 (93.3%) studies used measures of skeletal muscle mass, with dual energy X-ray absorptiometry (DEXA) being the most common modality (43.6%). Sarcopenia was found to be a consistent predictor of chronic disease progression, all-cause mortality, poorer functional outcomes, and postoperative complications. In conclusion, there is substantial evidence that sarcopenia impacts both medical and surgical outcomes. However, current research has utilized heterogeneous diagnostic criteria for sarcopenia. Further efforts to standardize the modalities used to diagnose sarcopenia in clinical research and practice will help strengthen our ability to study this important phenomenon.

## 1. Introduction

Sarcopenia, a form of muscle atrophy associated with aging and advanced disease, is an increasingly recognized occurrence in the medical literature [1]. While several definitions of sarcopenia exist, the most widely accepted criteria come from the European Working Group on Sarcopenia in Older People (EWGSOP) [1]. The group concluded that the diagnosis of sarcopenia requires clinical findings of low muscle mass and either low muscle strength or poor physical performance. Diagnostic testing is needed to confirm the presence of deficits in muscle mass, strength, and performance.

The prevalence of sarcopenia may be as high as 24% in patients aged 65 to 70, with older adults losing up to 15% of their total muscle mass during their 7th and 8th decade of life [2]. Despite increasing awareness of this phenomenon, no single definition for the diagnosis of sarcopenia has been universally adopted, and there is a growing but still modest understanding of the clinical implications of sarcopenia. Recent reviews, including those by Peterson et al. and Marty et al. [3,4], have begun assessing the prevalence of sarcopenia and its association with different disease states, although they have not collectively examined sarcopenia in studies of both surgical and non-surgical patient populations. In addition, while these reviews describe the general approaches that have been used to measure sarcopenia, they have not characterized how existing original studies on sarcopenia vary in terms of their diagnostic criteria for sarcopenia.

This investigation is a literature review of sarcopenia that examines the range of diagnostic criteria for sarcopenia and the quality of evidence for sarcopenia’s impact on clinical outcomes. While many diagnostic criteria have been utilized for sarcopenia, little research has been done to assess how many are in compliance with the EWGSOP criteria. This information is essential for understanding the evolution of sarcopenia as a disease process, especially given the amount of research published prior to the 2010 EWGSOP consensus. 

Not only is sarcopenia extremely common in the aging population, but it is also hypothesized to correlate with poorer prognosis in both the medical and surgical literature [2]. Therefore, a second aim of this review was to inform clinicians about the relevance of sarcopenia to individual medical and surgical specialties, and assess its association with important clinical outcomes including measurements of morbidity and mortality.

## 2. Methods

A literature search for primary studies was performed in the PubMed database by identifying the search term “sarcopenia” in the MeSH headings, title or abstract, or author-supplied keywords. Reference lists of systematic review articles were also screened for any additional studies missed during the initial search. No restrictions were placed on the year of study, with investigations dating from 1993 through 2015. Only studies in English were included.

A study was considered to meet inclusion criteria if it not only provided an operational definition for diagnosing sarcopenia, but also examined the effect of sarcopenia on any clinical outcome, either medical or surgical. If sarcopenic diagnostic criteria and outcomes were not clearly stated in the methodology, the study was excluded. All review articles, case reports, letters to the editor, editorials, and animal studies were also excluded. 

**Figure 1:**
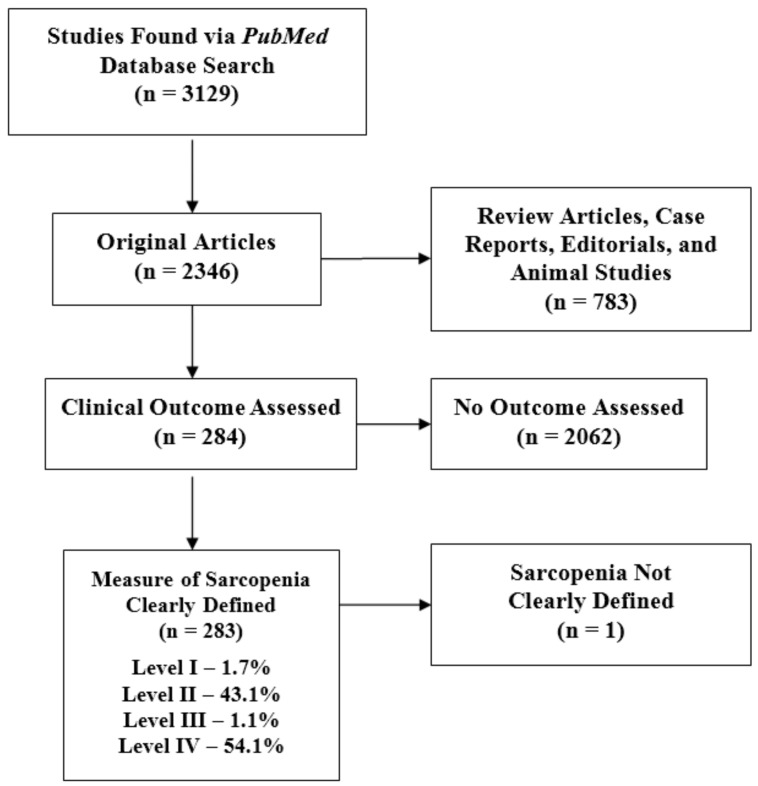
Flowchart of publications meeting inclusion criteria based on a PubMed search of the term “sarcopenia” with the level of evidence breakdown of the resulting studies.

Each potential article was reviewed by a minimum of two independent reviewers to determine appropriateness for inclusion. There was no discordance among reviewers regarding satisfaction of inclusion criteria. A total of 3129 studies were reviewed for inclusion. Of those studies, 283 satisfied the necessary inclusion criteria (Figure 1). Two separate reviewers assessed the field of medicine for each study based on thorough analysis of the target population, underlying patient pathology, and outcomes of interest. Levels of evidence were assessed using guidelines adapted from the Oxford Centre for Evidence-Based Medicine (Table 1). Two reviewers analyzed the methodological quality of each study before coming to a consensus on its level of evidence. Please refer to Supplementary Table S1 for a complete list of included studies with their information on sarcopenia diagnostic criteria, medical specialty, and level of evidence.

## 3. Results

**Figure 2:**
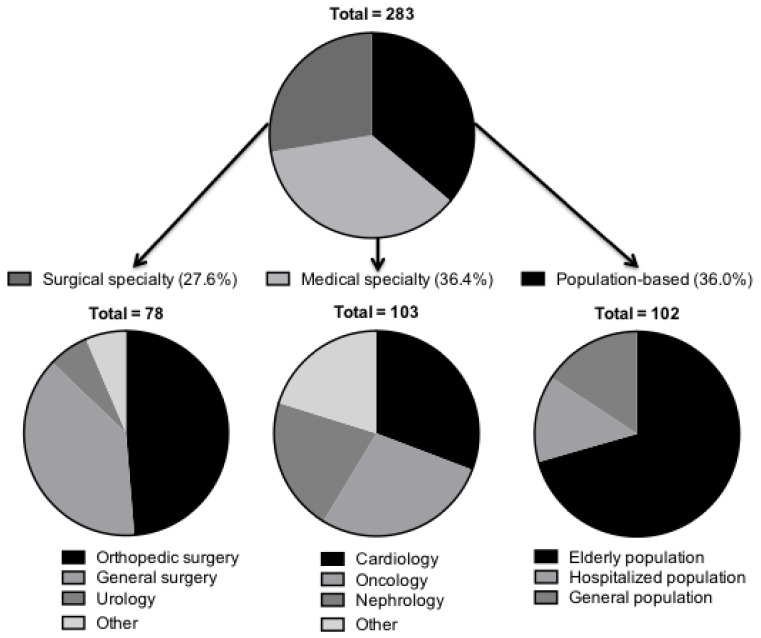
Distribution of included studies: surgical, medical, and population-based studies.

The number of annual research articles addressing sarcopenia has steadily increased since 1993 from 4 studies in 1993 to 666 in 2015 (Supplementary Figure S1). Of the 283 included studies, 102 examined the effect of sarcopenia on population-based clinical outcomes not specific to any medical field. Seventy-two (25.4%) of these focused on the elderly population, 14 (4.9%) investigated only hospitalized patients, and 16 (5.7%) studied the general population (Figure 2). In addition, 103 studies examined sarcopenia’s impact on outcomes in various medicine subspecialties. Among these, 32 (11.3%) studies examined sarcopenia in cardiovascular disease, 29 in oncology patients (10.2%), and 22 in kidney disease (7.8%), with the remainder examining sarcopenia in the context of hepatology, gastroenterology, pulmonology, and dermatology. In total, 78 studies examined sarcopenia in the surgical population, 49% of which were within orthopedic surgery, 38% in general surgery, and 6% in urology.

With regard to study design, cross-sectional studies accounted for 53.7% of all investigations, while prospective (20.5%) and retrospective (22.3%) clinical studies accounted for approximately one quarter each. Five randomized controlled trials have been performed. Five studies (1.7%) were level I investigations, 43.1% were level II, 1.1% were level III, and 54.1% were level IV studies. The median number of patients for all studies was 267.5 (interquartile range = 1378.3).

Studies varied in the criteria used to define sarcopenia. Overall, 264 (93.3%) studies utilized some measurement of skeletal muscle mass while 198 studies (70%) used skeletal mass alone to define sarcopenia. Methods of measuring muscle mass included dual energy X-ray absorptiometry (DEXA) in 43.6%, bioelectrical impedance analysis in 19.3%, and L3 total psoas index (defined as the total area of both psoas muscles taken on an axial cut of a computerized tomography (CT) scan at the level of the L3 vertebra divided by height squared) in 13.6% of studies (Table 2). Of the 40 studies (14.1%) using two criteria to define sarcopenia, the most common combination was a measure of muscle mass along with a test of grip strength (70%). 

A total of 27 studies (9.5%) used three functional criteria to define sarcopenia, most commonly adding a test of walking speed (66.7%) or a physical performance battery, such as the short physical performance battery (25.9%), to measurements of muscle mass and grip strength. Finally, only 6 studies (2.1%) used more than 3 criteria to define sarcopenia.

## 4. Sarcopenia in Medicine

### 4.1. Nephrology

Among end-stage renal disease (ESRD) patients, Kang et al. utilized Cox regression analysis and found an increased risk of death with sarcopenia with a hazard ratio (HR) of 1.89 in males (*p* = 0.003) and 2.20 in females (*p* = 0.001) (level II) [5]. In patients with renal cell carcinoma, sarcopenia is observed frequently (52.5%), leads to more dose-limiting toxicities (DLTs) for chemotherapy (*p* < 0.01, level II) [6], and is a predictor of longer length of hospital stay (1 additional day on average, *p* = 0.02, level II) [7]. Sarcopenia is also associated with increased number of metastatic sites (*p* = 0.006) and worsened overall survival (HR = 2.13, *p* = 0.016, level II) [7].

### 4.2. Hepatobiliary

Sarcopenia is independently associated with the development of hepatic encephalopathy in cirrhotic patients (*p* < 0.001, level II) [8]. In cases of hepatocellular carcinoma, sarcopenia leads to more DLTs with chemotherapy (82% versus 31%, *p* = 0.005, level II) [9]. 

### 4.3. Hematology/Oncology 

In studies of cancer patients irrespective of cancer type, sarcopenia is associated with severe chemotherapy toxicity events (25.5% versus 6.5%, *p* = 0.02, level II) [10]. For patients with breast cancer, sarcopenia is associated with a higher incidence of DLTs (50% versus 20%, *p* = 0.03, level II) and shorter time to tumor progression (101 days versus 173 days, *p* = 0.05, level II) [11]. Finally, sarcopenia is associated with higher mortality for pancreatic cancer patients (HR = 2.06, *p* = 0.006, level II) [12].

### 4.4. Cardiology

Hypertension occurs more frequently in sarcopenic patients (60.9% versus 49.7%, *p* = 0.002, level IV) [13]. Furthermore, sarcopenia in the obese population is independently associated with increased low-density lipoprotein levels and decreased high-density lipoprotein levels (OR = 2.82, 95% CI: 1.76–4.5, level IV) [14]. Sarcopenia is associated with reduced left ventricular ejection fraction (level II) [15], as well as with higher mortality in chronic heart failure (HR = 0.68, *p* = 0.017, level III) [16]. 

### 4.5. Pulmonology 

Sarcopenia was found to be present in 60% of patients with respiratory failure requiring mechanical ventilation (level IV) [17] and is associated with poorer forced vital capacity (OR = 1.99, *p* = 0.001, level IV) [18].

### 4.6. Population-Based

In hospitalized elderly patients, sarcopenia is independently associated with hospital acquired infection (relative risk of 2.1, *p* = 0.03, level II) [19] and all-cause mortality (HR = 1.32, 95% CI: 1.04–1.69, level IV) [20]. Similarly, in the non-hospitalized elderly population, sarcopenia has been independently associated with cognitive impairment, inability to perform activities of daily living, self-care, and community participation as assessed by the internationally applicable World Health Organization Disability Assessment Schedule (*b* = 3.01, 95% CI: 1.14–4.88, level IV) [21]. For these reasons, sarcopenia has been associated with institutionalization and greater healthcare demands, which subsequently contribute to estimated excess healthcare costs of $860 per male and $933 per female. The authors estimate potential savings of $1.1 billion per year with a 10% reduction in sarcopenia prevalence (level IV) [22].

## 5. Sarcopenia in Surgery

### 5.1. General Surgery

In an investigation of cost during the first post-operative year in patients undergoing elective surgery at a single institution from 2006–2011 (Michigan Surgical Quality Collaborative database), the difference in post-surgical costs between sarcopenic and non-sarcopenic patients was $16,455 at 30 days, with sarcopenic patients being more likely to be discharged to a rehabilitation facility or skilled nursing facility (*p* < 0.001, level II) [23]. In a study of 170 elderly patients undergoing emergency surgery, sarcopenic patients had more post-operative complications than non-sarcopenic patients (45% versus 15%, respectively, *p* = 0.005, level II) and higher rates of in-hospital mortality (23% versus 4%, *p* = 0.037), but there was no difference in length of stay [24].

### 5.2. Colorectal Surgery 

Sarcopenic patients had a significantly higher 30-day in-hospital mortality rate compared to non-sarcopenic patients (8.8% versus 7%, *p* = 0.001, level II) following colorectal cancer surgery [25]. Sarcopenia is also independently associated with poorer recurrence-free survival in patients undergoing surgery for resectable stage I-IV colorectal cancer (HR = 2.176, *p* = 0.01, level II) [26].

### 5.3. Transplant Surgery

Sarcopenia is an independent risk factor for mortality in patients following liver transplant surgery at both 1 year (OR = 0.53, *p* = 0.001) and 5 years (OR = 0.53, *p* < 0.001) postoperatively (level II), in addition to 1-year complications rates (OR = 0.67, *p* = 0.007) [27].

### 5.4. Hepatobiliary Surgery

Postoperatively, sarcopenia is an independent risk factor for decreased survival in patients undergoing surgery for hepatocellular carcinoma (HR = 1.52, *p* = 0.001, level II) [28]. Similarly, sarcopenia is associated with a longer hospital stay (39 days versus 30 days, *p* < 0.001) and increased rates of liver failure (33% versus 16%, *p* = 0.05) in patients undergoing major hepatectomy and extrahepatic bile duct resection for perihilar cholangiocarcinoma (level II) [29].

### 5.5. Orthopedic Surgery

Sarcopenia is associated with increased prevalence of multiple osteoporotic vertebral fractures (OVF) in women (OR = 2.56, *p* < 0.001, level IV) [30]. In obese patients, sarcopenia is associated with development of knee osteoarthritis when compared to obese patients without sarcopenia (OR = 3.51, level IV) [31].

### 5.6. Urology 

Sarcopenia is a useful prognostic marker for advanced urothelial carcinoma and an independent indicator of shorter overall survival in these patients (HR = 0.90, *p* < 0.001, level II) [32].

### 5.7. Gynecologic Oncology 

The presence of sarcopenia in patients undergoing surgery for endometrial cancer significantly impacts recurrence-free survival (HR = 3.99) but has not been shown to significantly impact surgical outcomes or overall survival rates (level II) [33].

### 5.8. Ear, Nose, and Throat

Sarcopenia is associated with decreased 5-year survival (66.7% versus 10.2%, *p* < 0.001) in patients with head and neck cancer receiving radiotherapy when compared to controls (level II) [34].

## 6. Discussion

Sarcopenia, a form of muscle atrophy associated with aging and advanced disease, occurs as part of the natural aging process involving involuntary loss of skeletal muscle mass and functionality. These age-related changes in body composition may have a genetic component, as genes involved in skeletal muscle mitochondrial function, oxidative capacity, and glucose uptake exhibit significantly decreased expression with aging [35]. Sarcopenia affects women and men equally and typically begins in the fourth decade of life, with an accelerated decline from the sixth decade onward [36]. Sarcopenia is an increasingly recognized and investigated phenomenon in the medical literature, with 4 studies examining its clinical effects in 1993 compared to 666 in 2015. As sarcopenia continues to be better understood as a disease entity, is essential that readers be aware of the composition of the literature in relation to the European Working Group on Sarcopenia in Older People criteria set forth in 2010 [1]. Despite the creation of a standardized definition of sarcopenia by the European Working Group on Sarcopenia in Older People requiring two positive criteria, low muscle mass and low muscle function (strength or physical performance), nearly 70% of investigations in this study define sarcopenia with muscle mass alone [1,2]. While muscle mass alone is a central component of sarcopenia, it is not the sole determinant of muscle function or strength, and the relationship between muscle mass and strength is not linear [1]. Thus, the EWGSOP definition requires fulfilling both diagnostic criteria because defining sarcopenia solely on the basis of muscle mass may be too narrow and limit its clinical value. 

Of the 40 studies using two criteria to define sarcopenia, the most common combination was a measure of muscle mass with a test of grip strength (70%). Fewer than 10% of studies used three functional criteria to define sarcopenia as outlined by EWGSOP. In future studies, standardization of sarcopenia assessment, particularly for the diagnosis of low muscle mass, will be crucial for clinical practice and intervention studies. The most commonly utilized techniques identified in this review were dual energy X-ray absorptiometry (DEXA), bioelectrical impedance analysis (BIA), and CT, each of which offers different advantages regarding validity and cost. CT and other advanced imaging including MRI are precise and have high validity, but are hospital-based, time-consuming, and costly [37]. DEXA is of intermediate cost and has good reproducibility but has been primarily used in the research setting, while BIA is inexpensive and portable but has sub-optimal validity. Future work to standardize the diagnosis of sarcopenia and utilize standardized methodology in clinical research would help improve our understanding and analysis of this phenomenon. Although it may be difficult to achieve a truly universal protocol for diagnosis of low muscle mass in sarcopenia, ultimately the technique used may vary based on cost, reliability, and practice setting, such as the use of BIA for community screening and interventions versus preoperative advanced imaging in patients undergoing surgery. In addition, future studies may investigate the use of novel serum biomarkers such as plasma transthyretin (TTR), which has been shown to be a surrogate marker of changes in lean body mass, as possible alternatives to imaging modalities for the diagnosis of sarcopenia [38].

This investigation revealed that sarcopenia has been found to be a predictor of chronic disease progression, poorer functional outcomes, and postoperative complications (both infections and non-infectious complications) across numerous medical and surgical specialties [7,8,9,10,11,12]. Sarcopenia has also consistently been shown to increase the cost of care and length of hospital stay [22]. Within oncology research, sarcopenia has been found to lead to more dose limiting toxicities and poorer disease free survival [9,10,11,12]. Among population-based research, sarcopenia is an independent predictor of all-cause mortality and cognitive impairment, increasing the chances of institutionalization later in life [23]. Sarcopenia has been found to be a predictor of poorer patient outcomes in not only the medical literature, but also in the surgical population. Among those diagnosed with colorectal cancer, sarcopenic patients had a significantly higher 30-day in-hospital mortality rate compared to non-sarcopenic patients [25]. Sarcopenia was also independently associated with poorer recurrence-free survival in patients undergoing surgery for resectable stage I–IV colorectal cancer [25]. Finally, sarcopenia has been found to be an independent risk factor for mortality in patients following liver transplant surgery at 1 year and 5 years. 

The mechanisms through which sarcopenia impacts patients across these diverse clinical settings are numerous and likely interrelated. Sarcopenia itself is a negative health outcome that is the multifactorial consequence of age-related changes, inflammation and disuse, and obesity and fat infiltration into skeletal muscle [39]. It has been postulated that the subsequent increased risk of physical limitation and disability negatively affects functional recovery, quality of life and independence in activities of daily living, and rehabilitation outcomes [40]. Other theories include the association of low muscle mass in sarcopenia with increased insulin resistance and risk of developing metabolic syndrome, as well as increased production of catabolic hormones and inflammatory mediators and cytokines [39]. This hypercatabolic and pro-inflammatory state triggers breakdown of muscle protein stores and is accompanied by increased levels of homocysteine [41], a hallmark of inflammatory disease states that has been associated with cognitive impairment and negative cerebro- and cardiovascular outcomes [42] and is postulated to be a risk factor for worsening sarcopenia [43].

Although sarcopenia has been less frequently studied in the surgical population, it is clear that sarcopenia is an important risk factor in clinical medicine and is associated with increased morbidity and mortality and worse outcomes in both non-surgical and surgical settings. Assessment of sarcopenia could not only help inform prognosis, but also guide optimal treatment for individual patients, such as exercise interventions and nutritional or physical therapy [44]. Sarcopenia may also prove useful for surgical risk stratification and identification of patients who would benefit from preoperative and postoperative intervention; one recent study in sarcopenic patients undergoing major surgery demonstrated that a novel clinical remediation program decreased payer and hospital costs as well as length of hospital stay [45]. This is not insignificant, as it is important to consider and address the demand sarcopenia has placed on healthcare systems, contributing to estimated excess healthcare costs of $860 per male and $933 per female [22]. The authors estimate potential savings of $1.1 billion per year with a 10% reduction in sarcopenia prevalence [22].

There are several potential limitations to this study. Over half of the reviewed sarcopenia research was from level IV evidence studies (54.1%), possibly limiting the strength of the conclusions that may be drawn. Additionally, while an effort was made to discuss studies with a higher level of evidence, a paucity of these studies exists within the surgical literature specifically. Furthermore, by including studies only available in English, data from the non-English literature, which may provide further insight into sarcopenia’s clinical effects and into researchers’ methodology worldwide, may have been missed. In addition, by limiting our search strategy to the term “sarcopenia”, studies that discuss muscle atrophy without using the specific term sarcopenia may have been excluded. Despite these limitations in the available data, it does appear that sarcopenia worsens clinical outcomes in aging patients. Finally, the majority of studies discussed here describe the impact of sarcopenia in the setting of disease. Future studies should assess the prevalence and long-term consequences of sarcopenia in healthy, free-living subjects, as sarcopenia affects both sexes indiscriminately beginning in the fourth decade of life and can occur in both physically active and inactive individuals.

## 7. Conclusions

While a large body of work supports the fact that sarcopenia is associated with poor clinical outcomes, current research has utilized heterogeneous diagnostic criteria for sarcopenia. Further efforts to standardize the modalities to diagnose sarcopenia for clinical research and clinical practice will help strengthen our ability to study this important phenomenon. Future prospective investigation will be beneficial to determine effective prevention and treatment strategies to minimize sarcopenia’s detrimental effects.

## Figures and Tables

**Table 1:** Levels of clinical evidence and study design, adapted from the Oxford Centre for Evidence-Based Medicine guidelines.

Level	Type of Evidence
I	Individual randomized controlled trials (RCTs)
	Systematic review of RCTs
II	Individual cohort studies
	Systematic review of cohort studies
III	Individual case-control studies
	Systematic review of case-control studies
IV	Cross-sectional studies
	Chart review
	Case series
V	Expert opinion
	Clinical observations
	Animal/bench research

**Table 2:** Commonly utilized measures of muscle mass used to define sarcopenia, examined by frequency.

	Definition	Percentage of Studies Utilizing
Dual Energy X-ray Absorptiometry (DEXA)	Uses X-ray absorption to provide an estimate of appendicular muscle mass	43.6%
Bioelectrical Impedance Analysis (BIA)	Approximates total body muscle and water composition by measuring resistance to electrical flow	19.3%
L3 Total Psoas Index (CT measurement)	Total area of both psoas at L3 vertebrae divided by height squared	13.6%
L4 Total Psoas Area (CT)	Total area of both psoas at L4 vertebrae	5.3%
L3 Psoas Area (CT)	Area of one psoas muscle at L3 vertebrae	3.5%
Total Quadriceps Area (CT)	Cross sectional area of both quadriceps muscles	1.8%
L3 Total Psoas Area (CT)	Total area of both psoas at L3 vertebrae	1.4%
Other	A definition of muscle mass used by less than three studies total	11.5%

## Supplementary Materials

The following are available online at http://www.mdpi.com/2077-0383/7/4/70/s1, Figure S1: Annual number of PubMed studies containing the search term “sarcopenia” from 1993 to 2015, Table S1: List of included studies with their information on sarcopenia diagnostic criteria, medical specialty, and level of evidence.
