# Supplementary material for: Diagnostic Criteria and Clinical Outcomes in Sarcopenia Research: A Literature Review
Source: J Clin Med. 2018 Apr 8;7(4):70. doi: 10.3390/jcm7040070 (PMC5920444; doi:10.3390/jcm7040070)
Supplement: Supplementary file 1 [file jcm-07-00070-s001.zip › jcm-286839-supl.-figure.docx]

**Supplementary Materials:**

**
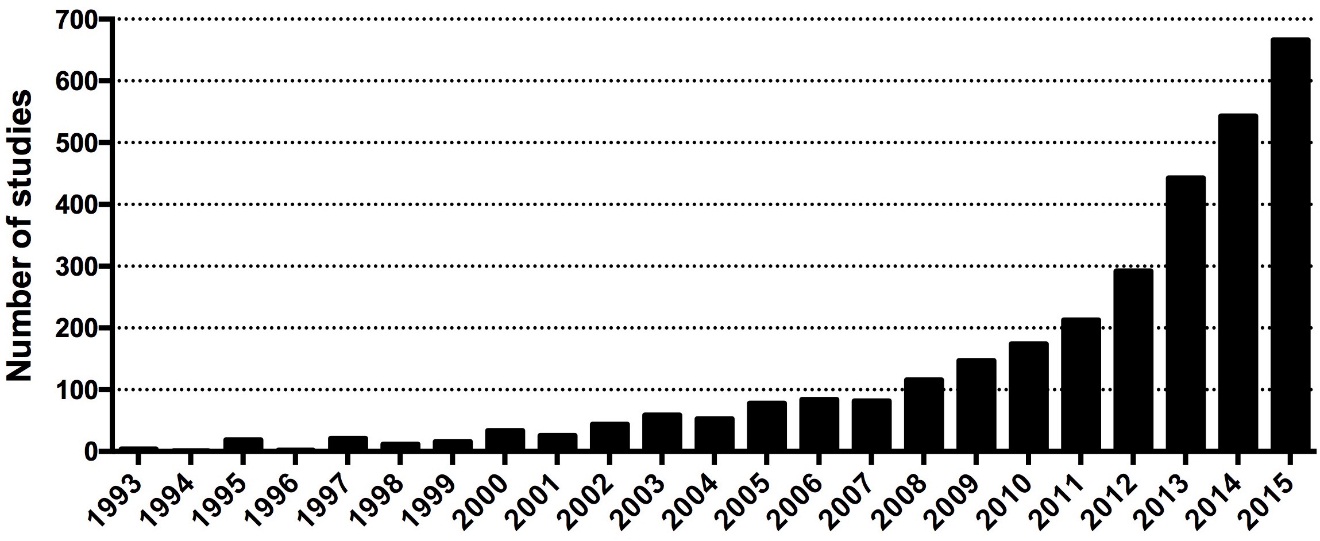
**

**Figure S1.** Annual number of PubMed studies containing the search term “sarcopenia” from 1993 to 2015.
